# Olive mill wastes: from wastes to resources

**DOI:** 10.1007/s11356-024-32468-x

**Published:** 2024-02-26

**Authors:** Ghizlane Enaime, Salahaldeen Dababat, Marc Wichern, Manfred Lübken

**Affiliations:** Institute of Urban Water Management and Environmental Engineering, Ruhr-Universität Bochum, Universitätsstraße 150, 44780 Bochum, Germany

**Keywords:** Olive mill wastes, Waste to recourses, Valorization, Energy, Soil amendment, Bioactive compounds

## Abstract

Olive oil extraction has recently experienced a continuous increase due to its related beneficial properties. Consequently, large amounts of olive mill wastes (OMWs) derived from the trituration process are annually produced, causing serious environmental problems. The limited financial capabilities of olive mills make them usually unable to bear the high costs required for the disposal of their wastes. Alternatively, the valorization of OMWs within the framework of the so-called waste-to-resource concept and their recycling can represent a successful strategy for the implementation of circular economy model in the olive industry, which could have significant socioeconomic impacts on low-income Mediterranean countries. There is, however, no unique solution for OMWs valorization, due to the wide variety of the wastes’ composition and their seasonal production. In this review, the potential of OMWs for being reused and the recent technological advances in the field of OMWs valorization are assessed. Special focus is given to the analysis of the advantages and limitations of each technology and to reporting the most significant issues that still limiting its industrial scale-up. The information collected in this review shows that OMW could be effectively exploited in several sectors, including energy production and agriculture. OMWs potential seems, however, undervalued, and the implementation of sustainable valorization strategies in large-scale remains challenging. More efforts and policy actions, through collective actions, encouraging subsidies, and establishing public–private collaborations, are still needed to reconcile research progress with industrial practices and encourage the large-scale implementation of the waste-to-resource concept in the olive sector.

## Introduction

With more than 3 million tons of olive oil produced annually, the olive oil extraction industry is considered as one of the most important agro-industrial sectors in the Mediterranean countries, contributing significantly to their economic and social development. Thanks to its nutritional value and content of bioactive compounds, olive oil is one of the most important food trends in the world and its demand is constantly increasing (Donner and Radic 2021). Olive oil is extracted either by traditional pressing or by three-phase centrifugal or two-phase centrifugal extraction processes. Although these processes may differ in the technology used and the quality of the extracted olive oil, they are similar in producing large amounts of wastes and by-products. It is assumed that the extraction of one metric ton of olive oil using three-phase systems produces in average 0.6 ton of olive mill solid waste (OMSW) and around 1.5 m^3^ of olive mill wastewater (OMWW). The use of the two-phase process, which is introduced as more ecofriendly extraction process, can reduce the amount of OMWW by 75%, but produces a semi-solid waste with higher moisture content (Markou and Georgakakis 2010). In overall, the total production of waste biomass generated from the extraction of olive oil is estimated to be at least 40 Mt/year, in which more than 20 Mt/year is corresponding to dry biomass (Di Giacomo and Romano 2022), while only OMWW annual production is estimated between 10 and 30 million m^3^/year (Annab et al. 2019).

**Fig. 1:**
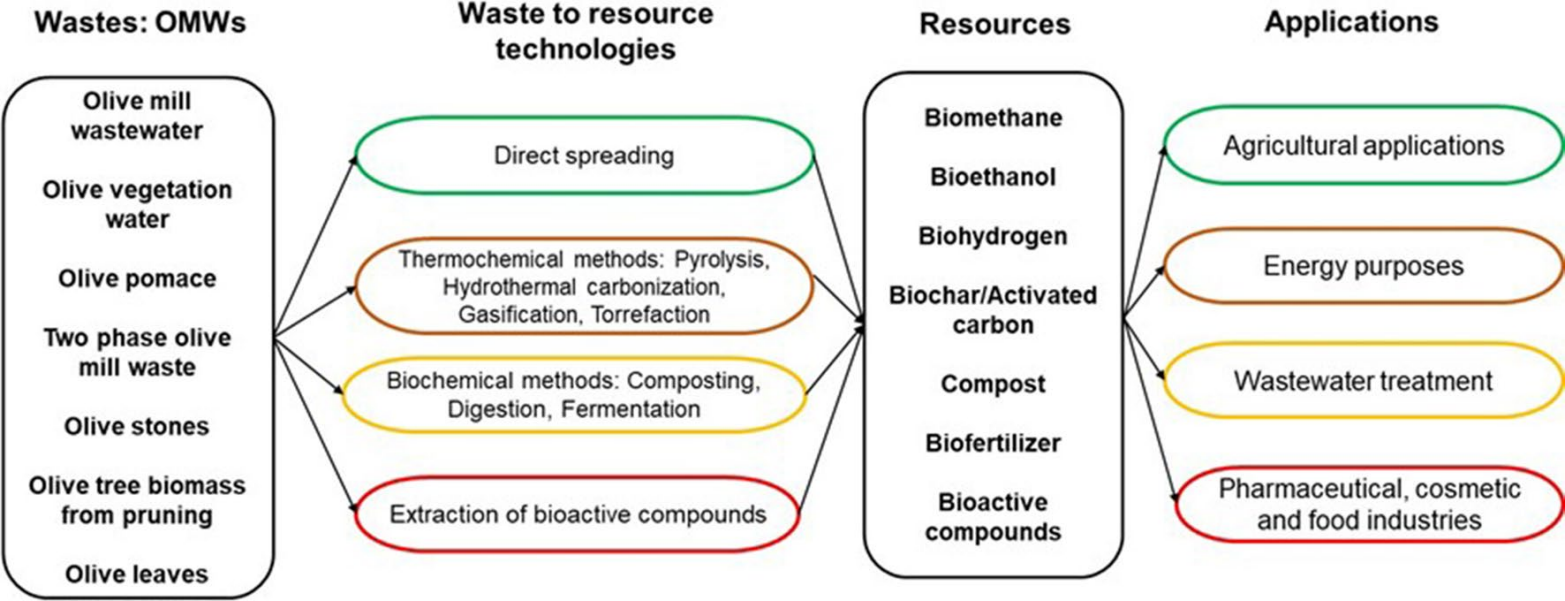
OMWs applications following the waste to resource concept

Olive mill wastes (OMWs) produced by the different olive oil extraction processes are characterized by undesirable color and odor, high acidity, high salt content, low alkalinity, and a very high organic load (Enaime et al. 2020a). OMWs are also characterized by a high content in phenolic compounds, which makes them phytotoxic and non-biodegradable (El-Abbassi et al. 2017). Until recently, these residues were considered undesirable due to their negative impact on the environment and the high costs required for their management and disposal. The management of OMWs is also challenging due to their seasonal production and the territorial dispersion of olive mills (Ntougias et al. 2013). The most common strategies adopted by olive oil producers are storage in open ponds or direct discharge into river streams for OMWW and use as fuel in co-incinerators for energy production for OMSW. Although these practices are relatively less expensive, they are however not considered as eco-friendly as they cause supplementary environmental issues such as soil and aquatic systems contamination, bad odors, high proliferation of insects and harmful methane emissions (Markou and Georgakakis 2010). Growing global awareness of the environmental challenges facing the world has increased the pressure to find sustainable alternatives to these traditional practices. The fact that OMWs are rich in organics and bioactive compounds, research is recently more oriented toward their valorization instead of their treatment using destructive methods, particularly with the promotion of the new approach of circular economy. OMWs are then no longer considered as a problem to be eliminated, but as a potential to be exploited (Fig. 1).

There is no exclusive solution for the valorization of OMWs. The choice of the appropriate recovery method depends on the properties of the waste, the local conditions, and the specific needs of olive oil producers. OMWs could be used in beneficial ways for bioenergy and biodiesel production in low-income countries, which will help reducing their need for foreign energy sources. These residues could also be used in many other applications, from the production of adsorbent materials for wastewater treatment to the extraction of valued antioxidants and the production of biofertilizer and soil amendments (Messineo et al. 2020; Uddin et al. 2021). OMWs can also be reused as growth medium for algae (Hodaifa et al. 2013b), as additive incorporated into construction materials (Hytiris et al. 2004), and to produce biopolymeric substance (Ntaikou et al. 2009). Despite the promising results obtained regarding the valorization of OMWs and their conversion into valuable resources, the large-scale application of the suggested methods is still limited for many technical and economic considerations. This contribution summarizes the key research investigations reporting on the valorization of OWMs and their use as resources instead of their treatment as wastes. Specific questions were as follows: What are the drivers and characteristics of the potential implementation of the new concept of wastes to resources in olive sector? What is the current knowledge related to this issue? What are the main limitations hindering the large adoption of this promising strategy and what are the future prospects?

## Olive mill wastes utilization techniques

Considering the big challenges facing today’s world, including high population increase, environmental pollution, and resources scarcity, new alternatives in the waste management sector that fit into the new concept of circular economy have recently being promoted. The circular economy, as previously defined, is an alternative business model which, unlike the linear model, consists of an intentionally regenerative industrial system, based on reusing materials that have reached their end of life and thereby increasing their value while reducing waste to a minimum (Murray et al. 2015). The large amount of OMWs generated annually has typically no industrial application and therefore constitutes a real challenge for olive mills. These wastes fall into the category of highly polluting and phytotoxic wastes and are therefore only considered from a treatment point of view. Pushed institutionally by the strength regulations and socially by the increased ecological awareness, olive oil producing industries are forced to start thinking about defining new directives for a transition towards circular economy models for a better management of their wastes. Within this concept, OMWs are considered as raw materials that could be directly used or converted into valuable products, allowing potential additional incomes for olive mill operators and making olive oil value chain more sustainable and environmentally friendly. In recent years, many efforts have been made to develop ways to effectively take advantage of these residues and recover their energy potential. Table 1 and the following sections will report the most important forms of recovery reported in the literature.

**Table 1:** Summary of different valorization methods of OMWs, the produced resources, and their applications

By-product	Valorization method	Produced resource	Application	Main findings	Reference
Olive pomace	Solvent extraction	Pomace oil	Food industry	Allow cooking at high temperatures	Donner et al. (2022)
Olive pomace	-	Flour and microparticles of olive pomace	Addition to chitosan-based films	-Significant improvement in antioxidant capacity -Improvement by 22% in tensile strength of films -Effectiveness in protecting nuts against oxidation for 31 days	De Moraes Crizel et al. (2018)
Olive pomace	-	-	Fortification of pasta with olive pomace	-Increase in phenolic compounds and antioxidant capacity of both cooked and uncooked pasta -Decrease in the optimum cooking time -Decrease in the rapidly digestible starch -Increase in the slowly digestible starch and resistant starch	Simonato et al. (2019)
Olive pomace	-	-	Adsorption of textile dye from aqueous solution	More than 80% of textile dye was removed	Akar et al. (2009)
OMSW	Anaerobic co-digestion of OMSW with microalgae (D. Salina)	-	Biogas production	Maximum methane (330 mL CH_4_/g VS) with a mixture of 75% OMSW–25% D. Salina and a C/N ratio of 26.7 has given the maximum methane yield	Fernández-Rodríguez et al. ( 2014, 2021)
TPOMW	Co-digestion of NaOH-pretreated TPOMW with food waste	-	Biogas production	The 20% NaOH-pretreated TPOMW caused the highest methane production of 503.6 mL CH_4_/g-VS	Al-Mallahi et al. (2016)
Olive stones	Drying grinding	Olive stones flour	Reinforcement filler of plastic materials	Enhancement in flexural strength and water barrier properties	Naghmouchi et al. (2015)
Olive leaves	Liquid–liquid extraction	Natural antioxidants extracts	Addition to sunflower oil, soybean oil, and their blend	Increase in the stability of the studied oils	Zahran et al. (2015)
Olive pomace and OMWW	Methanol extraction	Phenolic compounds	Enrichment of butter	High resistance against oxidative stress during storage	Mikdame et al. (2020)
OMWW and OMWW-impregnated biomass (sawdust and wood chips)	Convective drying and condensation	Recovered-condensed water	-	-95% water yield recovery -Low electrical conductivity and salinities -Suitable for reuse in agriculture after an additional treatment to adjust the pH and to decrease its COD content	Dutournié et al. (2019)
Olive Tree Pruning	Solvent casting method	Biodegradable packaging film	Food packaging biopolymer	-Increase in UV barrier up to 50% and in antioxidant capacity to 5.3% as compared to pure polyvinyl alcohol film -Improvement in tensile strength -Increase in thermal stability and reduction in water vapor permeability -Increase in barrier properties against UV, water vapor, and oxygen comparable to aluminum layer and plastic films	Sánchez-Gutiérrez et al. (2021)
Pruning biomass	Valorization in two processing plants: antioxidant plant (liquid–liquid extraction) and bioethanol plant (saccharification and fermentation)	Natural antioxidants and ethanol	Production of energy and high added value products	Ethanol (270 m^3^/day), antioxidants (33 t/day), electricity (self-sufficiency in the plant with a 58.1% surplus)	Romero-García et al. (2016)
OMWW and exhausted OMSW	Impregnation of exhausted OMSW with OMWW	Solid biofuel	Combustion	Increase in energy content and reactivity of exhausted OMSW following OMWW addition	Jeguirim et al. (2012)
OMWW	-	-	Alternative wetting agent	No significant difference observed for substrate containing 25% OMWW compared to control group	Kalmis et al. (2008)
OMWW	Combination of solar drying and composting	Organic fertilizer (57% organic carbon, 3.5% N, 1% P, 6.5% K) with low phenol content (2.9 g/kg)	Use as organic fertilizer for cultivation of pepper plants	Fertility similar to commercial NPK fertilizers	Galliou et al. (2018)
OMWW	-	-	Feed additive to a silage formulation for lambs	-Reduction in thiobarbituric acid reactive species and protein carbonyls -Increase in total antioxidant capacity, glutathione, and catalase activity in both blood and tissues -Improvement in animal productivity	Makri et al. (2018)
OMWW	-	Phenolic compounds	UV filters in sunscreens	Olive phenols are more active UV filters in a broader region of UVB and UVA	Galanakis et al. (2018)
OMWW	Ceramic membrane microfiltration	Polyphenols	Feed supplement for piglets	-Significantly increase in antioxidant mechanisms in blood and the majority of tissues -Decreased in oxidative stress in lipids and proteins	Gerasopoulos et al. (2015)
OMWW mixed with molasses	Fermentation	-	Ethanol production	Ethanol concentration and daily productivity values recorded at temperatures ≥ 20 °C (up to 67.8 g/L and 67.6 g/L day, respectively)	Nikolaou and Kourkoutas (2018)
OMWW	Fermentation of OMWW by *P. jamilae* (a Gram-positive bacteria isolated from OMWW based compost)	Exopolysaccharide (polymer)	Biotechnological interest due to its possible application as heavy metal biosorbent	Appreciable amount of extracellular polysaccharide (2.7 g/L) was produced with undiluted OMWW	Morillo et al. (2007)
OMWW	Yeast fermentation	-	Ethanol production	Maximum ethanol production (14.2 g/L) was obtained after 48 h of yeast fermentation using 50% diluted OMWW that was thermally processed and pretreated with *Pleurotus sajor-caju* cultivated on agar culture media of OMWW	Massadeh and Modallal (2008)
OMWW	Bacterial strain cultivation in OMWW	Bacterial cellulose	-	Enrichment of OMWW medium (%100) with yeast extract (5 g/L) and peptone (5 g/L) increased the amount of bacterial cellulose by 5.5 times	Sar and Akbas (2022)
OMWW	-	Natural dyes for textile industry	Dye bath for dyeing wool	Considerable color fastness properties for acrylic fiber	Haddar et al. (2014)
Olive vegetation water	-	Phenolic compounds	Fortification of milk beverages (similar to yogurt)	-	Servili et al. (2011b)

*VS* volatile solids

### OMWs as source of energy

#### Thermochemical methods

The use of thermochemical methods for energy utilization is mainly suitable for OMSW characterized by their lower water content, as compared to OMWW, and their considerable amount of energy (LHV = 15.58–19.81 MJ/kg), two important factors for considering a potential thermochemical utilization plant (Fokaides and Polycarpou 2013). OMSW has been directly used to produce heat and power through direct combustion. For instance, olive stones have been largely used as a substitute for fossil fuels in domestic boilers, in industrial plants or even in public heating systems (Pattara et al. 2010; López et al. 2010). Veraa et al. (2013) reported that a small-scale plant based on a downdraft gasifier fed with OMWs; small branches and leaves as well as crushed pits and a gas engine connected to the grid were able to provide 70 kW_electric_ and 110 kW_thermal_ as sanitary hot water needed in the olive oil extraction process with a biomass consumption of 105 kg/h. OMSW could be either combusted alone or in combination with other fuels having similar densities and energy contents such as coal (Niaounakis and Halvadakis 2006). Armesto et al. (2003) used effectively a bubbling fluidized bed in a pilot plant for the co-combustion of OMSW and two different coals, namely lignite and anthracite for energy generation purposes. The effectiveness of the co-combustion of various mixtures of olive cake with lignite coal was also confirmed by Atimtay and Topal (2004) using a circulating fluidized bed.

The direct application of OMSW for energy generation could be however associated to some limitations, mainly related to their moisture content (especially for two-phase olive mill waste; TPOMW), which increases packaging, storage and transportation costs and decreases the efficiency of the combustion process, while promoting a high CO emission (Enaime et al. 2022). The increase in CO emission is also observed due to the high content of OMSW in volatile matter. Topal et al. (2003) compared the combustion efficiency of olive cake and lignite coal treated separately and their co-combustion in a circulating fluidized bed. The results showed that CO and CH_4_ emissions are more important for olive cake than those for lignite coal due to its higher volatile matter content. Some operational problems like agglomeration, fouling, and corrosion could also occur as a result of the presence of impurities in OMSW, particularly alkali metals such as potassium and sodium (Enaime et al. 2022). To overcome these issues and effectively use OMSW as a biofuel, a processing step to upgrade their fuel properties prior to combustion is then required. Thermal conversion methods, like pyrolysis (Hmid et al. 2014), torrefaction (Benavente and Fullana 2015), gasification (Ducom et al. 2020), and hydrothermal carbonization (HTC) (Enaime et al. 2022), have been suggested to homogenize and convert OMSW into a form similar to coal. The application of these technologies allows a very significant reduction in volume, while effectively increasing the combustion efficiency of the produced carbon material as compared to the original biomass. Depending on the technology used, the distribution and properties of the resulting by-products vary. Choosing one method over another requires an in-depth knowledge of the nature and composition of the substrate and the intended application. For instance, TPOMW with high water content (> 70% water content) could be effectively treated by HTC without the need of energy intensive drying step. Benavente et al. (2017) confirmed that the hydrothermal conversion of TPOMW into hydrochar and its combustion with energy recovery resulted in net environmental savings for all impact categories with the exception of the impacts related to the untreated HTC process water. In another study, Volpe and Fiori (2017) used HTC to convert olive tree trimmings and olive pulp into hydrochar. The solid load proved to be a crucial parameter in determining the energy properties of produced hydrochars. The higher the solid charge, the higher the degree of carbonization, the yield of hydrochars and their energy content. In the same study, authors compared between the fuel properties of olive waste-derived char produced via HTC and low-temperature pyrolysis in a specific set of reaction conditions. Authors showed that HTC can be performed at a temperature 50 °C lower than that used in low-temperature pyrolysis in order to obtain similar biochar thermochemical properties. The fact that HTC is conducted at lower temperatures means that most of the starting mass is conserved, allowing for larger final yields as compared to pyrolysis chars (typically less than 60%) (Naisse et al. 2013). Interestingly, OMWW has also been used by Azzaz and co-authors as a feedstock for biofuel production via HTC (Azzaz et al. 2020). Authors claimed that HTC could be effectively applied to convert OMWW into a carbon-rich material with promising energy contents, up to 35.70 MJ/kg at HTC temperature of 220 °C. Conversely, Poerschmann et al. (2013) concluded that OMWW is not an ideal substrate for HTC treatment based on their low carbohydrates fraction, which allowed for low biochar yield production (30%, w/w). Alternatively, the co-hydrothermal treatment of OMSW and OMWW has been suggested. Volpe et al. (2018) successfully used HTC for the conversion of an olive waste stream mixture (olive pulp, kernels and OMWW) coming from a three phase-continuous process into a pelletized solid biofuel that showed high energy density and mechanical stability. Similarly, Enaime et al. (2022) applied HTC on olive stones previously used for OMWW filtration. The filter bed, exhibiting a moisture content of about 57%, was proved to be a good substrate for the production of solid biofuel (HHV = 31.0 MJ/kg) via HTC.

#### Biochemical methods

OMWs characterized by their high organic load especially in oil could be converted into various biofuels including methane, ethanol, and hydrogen using different biochemical techniques, such as anaerobic digestion and fermentation.

##### Methane production

The anaerobic treatment of OMWW for biogas production has been extensively documented in the literature. The produced biogas could be directly used for combined heat and power generation or its transformation into natural gas-quality bio-methane. The high organic load of OMWW and the presence of phenolic compounds seem, however, to affect the growth of many bacteria and to interfere with the activity of methanogenic consortia, thus limiting the effectiveness of any direct application of biological processes. One of the widely applied solutions to reduce the organic load of OMWW is their dilution, although this solution is a high-water consuming method and therefore generates a substantially larger volume of wastewater. Pretreatment with acidic, basic and saline chemicals; advanced chemical oxidation; and aerobic pretreatments are among many other pretreatment methods that have been also applied on OMWW prior to its use for methane production (Gunaya and Karadag 2015). Aerobic pretreatment has been effectively used to selectively attenuate the presence of phenolic compounds in OMWW. González-González and Cuadros (2014) reached a polyphenols removal efficiency of 56% after the first day of aerating OMWW by using indigenous microorganisms and a maximum removal of 90% by day 7. Aerobic pretreatments are, however, leading to a significant decrease in COD concentration, which reduces the potential for CH_4_ production in addition to the requirement for continuous and long-term aeration, leading to considerable energy consumption (Gunaya and Karadag 2015). A sustainable approach for the pretreatment of OMWW before anaerobic treatment has been investigated by Enaime et al. (2019), which consisted of the filtration of OMWW on olive stones followed by coagulation-flocculation as a second pretreatment step. This combined pretreatment process resulted in a total suspended solid and fatty matter depletion of about 82.5% and 73.8% and a simultaneous depletion of phenolic compounds and COD of about 11.3% and 23.2%, respectively. These results confirm the applicability of such an integrated system to enhance the treatability of OMWW for a further biological post-treatment as reported by Enaime et al. (2019).

OMSW has also been the subject of numerous investigations aiming at studying their anaerobic biodegradability and their biochemical methane potential. Borja et al. (2002) succeeded in operating a laboratory-scale reactor designed in digesting TPOMW under mesophilic conditions. The results showed that the daily production of methane increased linearly with the increase in organic loading rate (OLR). High removal efficiencies of COD and volatile solids (88.4 and 90.9%, respectively) and a maximum CH_4_ production rate of 2.12 l CH_4_/L day were achieved at an OLR of 12.02 g COD/L day, whereas the methane production rate decreased slightly when the OLR was increased from 12.02 to 15.03 g COD/L day. A linear increase in the CH_4_ production rate when the OLR increased from 1.5 to 9.2 g COD/L day with a COD removal efficiency ranging from 97 to 77% was achieved by Rincon et al. (2008) during the anaerobic digestion of TPOMW in a laboratory-scale continuous stirred tank reactor. The increase in the OLR was accompanied by a drastic decrease in the CH_4_ production rate. Authors noted a failure of the system when the reactor was operating at OLR higher than 9.2 g COD/L day. For both studies, an acclimatation step has been reported crucial to adapt the biomass to the nature of the TPOMW and improve its biodegradability and to enhance the methanization process.

The addition of nitrogen, an essential element for microbial activity, is necessary for the anaerobic treatment of OMWs to compensate for its deficiency. Co-digestion of OMWs with nitrogen-rich substrates, such as animal manures or slurries, municipal organic wastes, and agro-industrial wastes, was largely used to avoid nutrient deficiency and ensure an optimal C/N ratio, generally reported to be 20–30 (Maragkaki et al. 2018; Zheng et al. 2015). In addition to its ability to create an optimal balance among nutrients, trace elements, and pH, the co-digestion of OMWs and other feedstocks could be considered as an attractive management method as it offers the possibility of simultaneously processing two or several feedstocks in one reactor. In addition, it allows the dilution of toxic compounds and inhibitors present in OMWs, hence improving the stability of the process and the production of biogas (Mata-Alvarez et al. 2014; Shah et al. 2015). Azbar et al. (2008) reported an enhancement of biogas production by about 90% when OMWW was mixed with laying hen litter, while 22% more was achieved when mixed with cheese whey. Interestingly is the co-digestion of OMWW with OMSW as both streams are a problematic issue for olive mill operators and OMSW is a substrate rich in nitrogen (TKN = 10–20 g N/kg total solid) that could be used to compensate for OMWW lack in alkalinity and ammonium. Boubaker and Ridha (2007) investigated the anaerobic co-digestion of OMWW with OMSW in laboratory-scale using tubular digesters operated at mesophilic temperatures and fed semi-continuously at OLRs varying from 0.67 to 6.67 g COD/L day.

Higher methane yield was recorded when OMWW was co-digested with OMSW compared to that observed when OMWW was digested alone. The highest CH_4_ production of about 0.95 L/L day was reached at an OLR of 4.67 g COD/L day, while the maximum COD removal efficiency of 89% was achieved at the lowest OLR of 0.67 g COD/L day.

##### Ethanol production

OMSW could be a promising substrate for ethanol production due to their high cellulose and hemicellulose content (Battista et al. 2016; Fernandes et al. 2016). OMSW is, however, also rich in lignin known for their inhibitory effect on cellulase enzymes’ activity and saccharification process, which can reduce the efficiency of its conversion to ethanol (Ximenes et al. 2011). Several pretreatment methods including physical (e.g., microwave, ultrasound), chemical (e.g., alkali, dilute acid, ozonolysis, organosolvents) or physicochemical (e.g., steam explosion, hydrothermolysis, and wet oxidation) methods have been used in single or in combined processes to remove lignin from OMSW (Aguilar-Reynosa et al. 2017; Rosen et al. 2019; Peretz et al. 2021). The selection of a pretreatment method should be based primarily on its ability to provide enzymes access to cellulose allowing, hence, high concentrations of fermentable sugars, but should also be cost effective and limit the formation of fermentation inhibitors. Tayeh et al. (2020) reported that microwave treatment of OMSW in the presence of formic acid allowed highest saccharification rates (90% of cellulose fraction hydrolysis and 91.5 mg ethanol per gram OMSW) and important fermentation yield (15.9 g/L ethanol), while microwave with water treatment resulted in less saccharification and ethanol production (9.6 g/L). Fernandes et al. (2016) exploited the fermentation of extracted olive pomace after dilute acid hydrolysis pretreatment for bioethanol production. Although the adopted pretreatment significantly removed hemicellulose, the subsequent enzymatic treatments showed that the pretreated biomass still exhibited a significant recalcitrance to cellulase action, when compared to the equivalent material pretreated by autohydrolysis. A size reduction and an alkaline post-treatment of extracted olive pomace were tested for the improvement of enzymatic saccharification. The result showed that size reduction was more effective in reducing the lignin content and improving the enzymatic accessibility as compared to the alkaline post-treatment. In a study performed by Najafi et al. (2021), an integrated process regrouping fermentation, anaerobic digestion of fermentation residues, and lignin production was performed. In the same, study three different pretreatments have been tested, namely liquid hot water, organosolv, and acid-catalyzed organosolv. By using this combined process, authors reported a production of 295.5 L bioethanol, 137.2 m^3^ biomethane, and 347.1 kg lignin. The anaerobic treatment of waste streams resulting from ethanol production to provide biomethane and lignin improved the energy recovery of the whole plant up to 2.5 times as compared to single-product plants. Organosolv and acid-catalyzed organosolv have been reported improving ethanol and methanol production. Interestingly, the developed integrated process allowed an overall energy production per hectare of olive trees of about 521.6 L gasoline.

Ethanol production from OMWs could be a promising alternative to fossil energy sources. However, the production process was not found to be profitable despite the availability and low cost of raw materials (Christoforou and Fokaides 2016). Additional efforts are still needed to optimize the fermentation process and improve its efficiency in order to make ethanol derived from OMWs a more competitive product.

##### Biohydrogen production

OMWs, either fresh or stored, have also been reported as suitable feedstocks for biohydrogen production due to their content in organic substances, such as sugars, tannins, polyalcohols, pectins, lipids, and organic acids (Padovani et al. 2013). Biohydrogen is mainly produced by dark-fermentation or photo-fermentation or a sequential operation of both processes. Scoma et al. (2013) used a mesophilic packed bed biofilm reactors filled with ceramic cubes and inoculated with an acclimated acidogenic microbial consortium for biohydrogen production from dephenolized olive mill wastewater. The results showed that biohydrogen relative amount and productivity increased from 3 to 32% and from 0.20 to 6.10 dm^3^/m^3^ h, respectively, by decreasing the HRT from 7 to 1 day. In another study, Eroğlu et al. (2006) introduced two novel two-stage processes for hydrogen production from OMWW. In the first two-stage process, dark fermentation by activated sludge cultures followed by photofermentation process by *Rhodobacter sphaeroides* is involved leading to a promising hydrogen production potential of about 29 L H_2_/L OMWW. The hydrogen production using the second two-stage process, which involved a clay treatment step followed by photofermentation by *R. sphaeroides* O.U. 001 allowed a higher hydrogen production (35 L H_2_/L OMWW) and a promising COD conversion efficiency (52%). The results showed that both pretreatment processes enhanced the photofermentation process leading to promising hydrogen production even with highly concentrated OMWW. The simultaneous production of bioethanol and biohydrogen from OMWW-olive pomace mixture using *Saccharomyces cerevisiae* anaerobic fermentation was also investigated by Battista et al. (2016). Authors studied several pretreatments (ultrasonic pretreatment, basic pretreatment, and calcium carbonate addition) to improve glucose release and then enhance bioethanol and biohydrogen production and simultaneously reduce the inhibiting effect of polyphenols on the fermentation process. The results showed that all the pretreatment methods improved bioethanol and biohydrogen production yields, with basic and ultrasonic pretreatments resulted in the highest bioethanol and biohydrogen concentration, due to their contribution in improving the hydrolysis of lignin and cellulose and in increasing the soluble sugars (in particular glucose) content in the reaction mixture.

### Application of OMWs in agriculture

#### Direct spreading of OMWs in soil

The use of OMWs for agricultural purposes has received a great deal of attention as a potential method for their valorization. Among several application routes, direct spreading of OMWs in soil was widely discussed, between those who supported their beneficial effect excluding any significant risk for crops and soil properties and those who showed negative effects in particular on surface and groundwater. The difference in the obtained results may be attributed to the difference in the experimental conditions used in each study, such as differences in the spreading method and doses, the type of soil and phonological stage of the crop and the climatic conditions. For optimal OMWs spreading that respect specific regulations, a proper amendment method and a rational use of OMWs should be applied. Regarding the spreading of OMWW, it has been reported that nitrogen (almost exclusively in organic form), potassium, phosphorus, magnesium, and organic matter present in OMWW can substitute some of the nutrients provided with chemical fertilizer and improve soil fertility (Regni et al. 2017). It was estimated that approximately 3000–6000 kg of dry organic matter, 25–50 kg of nitrogen, 15–30 kg of phosphorus, and 80–160 kg of potassium could be provided by spreading 80 m^3^ OMWW/s in soil (Di Giovacchino and Seghetti 1990). Fertigation with OMWW is preferably applied on clay–loam soils, characterized by their high cation-exchange capacity (Regni et al. 2017). OMWW has also been shown to increase the exchangeable potassium in a loamy clay soil and phosphorus in a red soil (Gioffré et al. 2004). OMWW spreading can cause slight changes in the soil pH due to its acidic character, which can also affect the mobility of macro- and micronutrients and the soil microbiological activity (IOOC 2004). However, the effect of OMWW spreading on soil pH was reported significantly reduced after few months, due to the production of ammonia resulting from the bacterial breakdown of OMWW organic matter and the buffering capacity of soil (Regni et al. 2017). The effect of OMWW spreading on soil microbiology is due not only to the variation in the soil pH, but also to the contribution of OMWW organic matter, which induces an increase in the soil microflora. Mekki et al. (2006a) reported a significant increase in soil actinomycetes, spore-forming bacteria and soil fungi and a significant reduction in the number of soil nitrifying bacteria. Similarly, Mechri et al. (2007) observed an increase in soil fungi, Gram-negative bacteria and actinomycetes after the addition of more than 30 m^3^ OMWW/ha and a significant decrease of Gram-positive bacteria after 1 year of OMWW application in a field of olive trees. Although the magnitude of the effect of OMWW application on soil microflora is different between different investigations, it is generally reported that OMWW amendment caused minor long-term effects on soil microflora and that no evidence of any inhibitory effect on the growth of soil microorganisms was recorded.

The application of OMWW as soil amendment has been reported as negatively affecting cultivated plants; this effect is mainly associated to OMWW application close to the sowing period and to the used dose (Bonari et al. 2001). For instance, an inhibition of wheat during the early stages of growth was observed, while no adverse effects were noticed at harvest (Boz et al. 2003). The toxicity of OMWs toward plants depend also on which part of the plant is in contact with OMWs. Tomato roots have been reported more sensitive to OMWs than tomato shoots (Ouzounidou et al. 2008). These effects may be associated to the lipophilicity of OMWs fatty acids and phenols, which limits the accessibility of nutrients inside the biological membranes (Saadi et al. 2007). The identification of phenolic compounds responsible on OMWs phytotoxicity was the subsect of many research studies. Isidori et al. (2005) while studying the effect of main OMWs phenolic compounds on watermelon, garden cress, and sorghum germination reported hydroxytyrosol and catechol as the responsible on the highest observed phytotoxicity effect. The same results were also reported by Aliotta et al. (2002) in regard to the germination of radish and durum wheat seeds. A dose-dependent phytotoxic effect of phenols on the germination of English cress and tomato was stated by Greco et al. (2006). In another study, Enaime et al. (2020a) showed that the phytotoxicity of OMWW anaerobically treated appears to be determined not only by the monomeric phenols but also by other toxic components unaffected by the anaerobic treatment.

OMSW spreading on agricultural surfaces has also been adopted as an operationally simple and economically feasible valorization method. The composition of pomace is relatively similar to that of organic amendments, and thus can be used for agronomic purposes (Toscano and Montemurro 2012). Other than being economical and easily practicable management method for olive mill operator, it has been reported conferring considerable benefits for soil characteristics and crops productivity. Kavdir and Killi (2007) demonstrated that soil amendment with pomace improves its water holding capacity, structure, and stability, making it less susceptible to erosion. The incorporation of olive pomace in soil increases its organic matter and enhanced its biological activity, without detectable negative effects on pH value and salinity (Regni et al. 2017; Innangi et al. 2017). The effect of pomace application on soil organic matter is strongly depending on the application method. Nasini et al. (2013) proceeded to the spreading of large amounts of pomace (50 t/ha) in an olive grove in central Italy for 4 consecutive years and reported an increase in organic matter content, total nitrogen, exchangeable potassium and magnesium, and available phosphorus and a slight decrease in pH. Soil amendment with olive pomace could be a valuable method to improve its fertility with costs lower than those required when using chemical fertilizers. Brunetti et al. (2005) observed in a field experiment an important increase in soil total nitrogen after 2 years of olive pomace application. Similarly, Proietti et al. (2015) reported a significant increase in soil available phosphorus in the first 15 cm of the treated soil; however, no effect was occurred in the 15–30 cm layer as compared to the control. Regarding the effect of olive pomace amendment on soil microbiology, Nasini et al. (2013) reported that olive pomace could be only a short-term substrate for soil microbiota recording no significant increase in the number of total microorganisms following the application of pomace. Authors also observed no difference between treated soil and the control when olive pomace is applied at the 15–30 cm dept, suggesting that environmental concerns related to the use of pomace as a soil amendment can be excluded considering that upper layer is not affected by olive pomace spreading. Soil amendment with olive pomace did not only affect soil properties but also crops productivity (Nasini et al. 2013). The application of wet olive pomace on a maize crop for 2 years allowed an increase in the production yield, grain gross protein, grain soluble carbohydrate content, and yield and number of grains per corncob (Tajada and Gonzalez 2004). An increase in the yield of winter wheat crop grown in greenhouse of up to 198% has also been observed by López-Piñeiro et al. (2006). The application of olive pomace as soil amendment on olive trees was also studied. Proietti et al. (2015) applied wet olive pomace on olive grove for 3 consecutive years and observed an increase of olive trees productivity without exhibiting a negative impact on olive oil quality. An enhancement in fruit growth and yield was also reported by Nasini et al. Nasini et al. (2013) when applying olive pomace on olive trees during 4 years without negative effects on the oil content. The positive effect of OMWs spreading on soil and crops is conditioned by the respect of the maximum spreading load and the correct spreading technique and time. López-Piñeiro et al. (2007) reported that the optimal spreading period is corresponding to the period before the resumption of vegetative growth. The spreading load could be higher when OMWs are applied on tree crops such as olive tree. Additionally, the nature of OMWs is also an important factor in determining the spreading load. In Italy, the agronomic use of OMWW derived from traditional extraction systems is allowed up to 50 m^3^/ha annually, while up to 80 m^3^/ha is allowed when OMWW is derived from modern extraction systems (Regni et al. 2017).

#### OMWs composting

Composting of OMWs, which allows the breakdown of labile organic compounds and the production of a material rich in organic matter, macro and micronutrients and free of phytotoxic elements, was reported beneficial for plant growth and effectively facilitate the integration of OMWs as organic amendment into the soil (Alburquerque et al. 2006). Co-composting of OMWs with other agricultural wastes, such as olive tree leaves, straw, cotton gin waste, grape stalks, and animal manures, has also been studied (Majbar et al. 2018; Cayuela et al. 2004). Compost and co-composts derived from OMWs have been effectively used as organic fertilizers for horticultural crops (Alburquerque et al. 2006), olive trees (Cayuela et al. 2004), and also as part of the substrate or growing media for ornamental plant culture (Garcia-Gomez et al. 2002). In Spain, compost from wet olive pomace is included, as an organic amendment, in the legislation on fertilizing products, which also specified the physico-chemical requirements of the product (Pardo et al. 2017). Although the results showing that composts derived from OMWs exhibited a satisfying degree of humification and no phytotoxic effect, OMWs themselves could be, however, difficult substrates for composting, due to their high moisture content (> 90% in OMWW) and to the presence of compounds, like fats and polyphenols, that exhibit an antimicrobial character (Ramos-Cormenzana et al. 1995). Moreover, the dense and sticky physical texture of OMSW especially TPOMW makes sometimes the aeration process hardly performed, which can lead to the formation of aggregates (Marks et al. 2020). This limitation has led to search for more cost-effective alternatives for the efficient application of OMWs in soil.

#### Application of OMWs-derived biochar/hydrochar as soil amendment

Conversion of OMWs into more stable carbon material before its application as soil amendment has been introduced as an alternative for their valorization. Pyrolysis of agro-residues and their conversion into biochar can fulfil the aim of closing the loop in agriculture and circular economy objectives in Mediterranean countries. Biochars generally produced by slow pyrolysis can be used to increase soil pH, electrical conductivity, available P and cation exchange capacity (Biederman and Harpole 2012; Marks et al. 2016). The alkaline character of biochar, their elevated concentration in Ca and Mg, and their sorption capacity can make them useful for remediation strategies, e.g. metal retention (Alburquerque et al. 2014). The application of biochar derived from OMWs to soil showed positive effects on plant growth by promoting the proliferation of fine root and facilitating water and nutrients retention (Olmo et al. 2014; Marks et al. 2020). OMWs conversion to biochar is also a good strategy for carbon sequestration, due to its high concentration in recalcitrant carbon showing low mineralization rates and long residence times to degradation; this capability is more pronounced for biochars derived from OS (Alburquerque et al. 2014; Olmo et al. 2014). As most lignocellulosic feedstocks, OMWs-derived biochars are, however, exhibiting low nitrogen contents (Table 2), which could limit its efficiency as fertilizer (Marks et al. 2020). Haddad et al. (2017) proceed to the impregnation of wood sawdust with OMWW in order to recover their nutrients content, followed by drying and slow pyrolysis. This strategy does in fact increase nitrogen content of the final biochar in addition to enriching it with macro- and micronutrients. OMWs-derived hydrochars are also valuable carbonaceous materials in agriculture for their slow carbon mineralization and high mineral contents (Kambo and Dutta 2015). Although biochar and hydrochar can be used in agriculture for similar purposes to improve soil structural, physico-chemical, and microbiological properties, their physicochemical properties are different (Table 2), because the reactions involved during the thermal process used for their production are different (Enaime et al. 2023). Hydrochar is generally exhibiting low pH as compared to OMWs-derived biochar (Table 2), due to the presence of organic acids generated during the reactions of dehydration and decarboxylation, which makes it more suitable for alkaline soils to lower their pH (Khosravi et al. 2022). Hydrochar is also characterized by a high density of phenolic, carboxylic, and aliphatic groups, in contrast to biochar produced by pyrolysis having few aliphatic groups and more aromatic structures, which makes of hydrochar more easily decomposable compared to biochar (Azzaz et al. 2020; Gascó et al. 2018). Moreover, the HTC liquid phase is a concentrate of some nutrients such as potassium but has so far not received an attention as a fertilizer (Marks et al. 2020).

**Table 2:** Main characteristics of OMWs and their derived compost, biochar and hydrochar (Tarf et al. 2022; Pardo et al. 2017; Gigliotti et al. 2012; Michailides et al. 2011; Peña et al. 2022; Fornes et al. 2015)

Parameter	OMWs	Compost	Biochar	Hydrochar
pH	4.2–6.8	7.4–9.5	7.7–11.0	5.29
EC [dS/m]	0.98–12.0	1.6–7.3	0.28–2.15	0.29
TN [g/kg]	0.61–18.5	11–54.0	4.1–13.0	1.91
C/N	28.2–72.9	9–36.0	26.17–227	29.52
Total P [g/kg]	0.06–2.2	0.2–30.0	0.14–8.34	0.04
Total K [g/kg]	1.97–29.7	2.3–44	6.3–30.0	1.98
TOC [g/kg]	34.2– 539.0	382–580	483–933	-
Phenols [%]	0.5–10.7	0.1–3.8	-	-

#### Application of OMWs as biopesticide

Due to their phytotoxic and antimicrobial properties, OMWs have also been tested in agriculture as biopesticide for crops protection and as herbicide for weed control (Larif et al. 2013; Lykas et al. 2014; Boz et al. 2009). Boz et al. (2009) showed that olive pomace could be applied at a rate of 10 to 20 t/ha for adequate weed control and crop safety. OMWW was also used as an efficient alternative to commercial corrosive disinfectants such as sodium hypochlorite, due to its high content on phenolic compounds such as gallotannins, p-coumaric acid, and hydroxytyrosol, known for their antimicrobial effect and inhibiting effect on germination, growth, and development of different weeds (Doula et al. 2012; Lodhi 1976). A hydroxytyrosol-rich OMWW and a hydroxytyrosol-rich extract from fresh OMWW showed high fungicidal activities against *Verticillium dahliae* (Yangui et al. 2010). Compared to untreated plants, the incorporation of hydroxytyrosol-rich OMWW and hydroxytyrosol-rich extract into soil significantly reduced *Verticillium* wilt disease incidence by 86 and 83% and wilt severity by 86 and 84.5%, respectively. In another study, Yangui et al. (2009) observed an enhancement of germination percentage, root length, shoot height and shoot weight of tomato and muskmelon seed plants after the addition of hydroxytyrosol rich concentrate as a natural disinfectant at 10% (dw/v). The allelopathic effects of sterile water extracts of TPOMW and TPOMW composts on seed germination of highly invasive weeds, namely *Amaranthus retroflexus* L., *Solanum nigrum* L., *Chenopodium album* L., and *Sorghum halepense* (L.) Pers., have been studied by Cayuela et al. (2008). The results showed a substantially inhibition of *Amaranthus retroflexus* L. and *Solanum nigrum* L. germination by both TPOMW and immature TPOMW compost extracts, while an only partially reduction in the germination of *Solanum nigrum* L. using mature composts extracts has been observed. The authors claimed that the fungicidal capability of OMWs mature composts could be due to the presence of toxic metabolites (antibiosis) produced by some microbial communities during the composting process. In the same study, a significant hatch suppression was also showed by TPOMW composts, which was supposed to be due to the existence of bioactive compounds able to pass through the nematode eggshell. The use of OMWs-derived composts as a soil additive could also exert a biopesticidal action against plant pathogens due to their high content of nutrients and biocidal compounds (tannins and phenolic compounds), which stimulate root development and boost plant growth (Sasanelli et al. 2011). Oka and Yermiyahu (2002) suggested that the nematode suppressiveness of composts may be due to their high N–NH_4_ concentrations and high electrical conductivity values.

#### Livestock feeding with OMWs

Among the applications of OMWs in agriculture, the incorporation of OMWs into the diets of livestock has been also suggested as a strategy to minimizing both the costs related to OMWs management and those related to animal feeding, as animals become less dependent on conventional feeds such as cereal grains (Molina-Alcaide and Yáñez-Ruiz 2008). Researchers focused their studies to find alternative ways to reduce the content of saturated fatty acids in milk and meat due to their hypercholesterolemic and thrombogenic effects that could lead to cardiovascular diseases. The enrichment of diets with agro-industrial by-products, such as OMWs, has been reported as beneficial due to their high content in unsaturated fatty acids (Tzamaloukas et al. 2021). Molina-Alcaide et al. (2010) reported that the feeding of ruminants with olive pomace at 10% reduced the overall feeding cost and improved milk composition. In another study, Nasopoulou and Zabetakis (2013) reviewed that olive pomace could be exploited as an alternative dietary lipid source in compounded fish feeds for aquaculture and could also be incorporated in moderate consumption in animal feeds, without affecting animal performance while reducing saturated acids and enriching meat and milk with unsaturated fatty acids. Other investigations reported that the level of cholesterol and saturated fatty acids decreased and those of unsaturated fatty acids increased in egg yolk after OMWs incorporation (Afsari et al. 2014; Abd El-Samee and Hashish 2011). OMWW has also been used as a source of polyphenols additives to increase the antioxidant defense of productive animals. Several studies have been carried out to understand the effect of their addition to the diet of different animals such as broiler chickens and mammals on their antioxidant defense. Gerasopoulos and Petrotos (2022) analyzed the distribution of fatty acids in plasma and tissues of piglets alimented with feed containing polyphenols extracted from OMWW. The addition of polyphenols to the piglets feed allowed a decrease in omega-6/omega-3 ratio, which leads to more omega-3 fatty acids in the meat of pigs. In another study, Gerasopoulos et al. (2015) incorporated prefiltered OMWW into broilers’ feed and examined their antioxidant activity. The 24 broilers receiving feeds supplemented with OMWW were observed for 37 days, and bloods and tissues (muscle, heart, liver) samples were collected at different periods. Higher total antioxidant capacity in plasma and tissues was observed for broilers receiving feeds supplemented with OMWW along with a significantly lower protein oxidation and lipid peroxidation levels as compared to the control. Even with the well-documented benefits of OMWs addition in animal feeds, there are some barriers that limit their wider use, including their low content on protein and their counterproductive effect due to their high energy content, which can reduce the animals’ total feed intake. The respect of the optimal addition percentage (10% of total diet at most, although 5% is usually recommended) is then necessary to avoid any side effect and guarantee a balanced diet (Berbel and Posadillo 2018).

### Application of OMWs for wastewater treatment

Biosorption has been proven as an effective and economically competitive process for wastewater treatment, especially when the biosorbent is derived from biowastes and agricultural by-products. Olive crops, which cover a global cultivated area of approx. 10 million hectares, are sources of huge amounts of low-cost and locally available waste materials that can be either directly exploited as biosorbent of organic and inorganic contaminants or used as precursors for biosorbents preparation. OS were effectively used for the biosorption of Alizarin Red S and methylene blue dyes from aqueous solution allowing maximum adsorption capacities of 16.10 mg/g and 13.20 mg/g, respectively (Albadarin and Mangwandi 2015). The authors reported that the adsorption rate is controlled by film diffusion in addition to the implication of adsorption mechanisms such as ion exchange and chelation. The ability of crude OS to adsorb iron from industrial wastewaters was also studied by Nieto et al. (2010). The equilibrium adsorption capacity was higher when the particles size decreased from 4.8 to < 1 mm. The percentage of iron adsorption increased from 30 to 70% when the initial concentration of biomass increased from 25 to 125 g/dm^3^. The optimum concentration of OS was fixed at 37.5 g/dm^3^. In another study (Banat et al. 2007), olive pomace after solvent extraction have been used in combination with charcoal produced from OMSW as an adsorbent for the removal of methylene blue from aqueous solutions in batch and fixed bed experiments. Batch experiment allowed to reach a dye removal efficiency of up to 80% using a sorbent concentration of 45 mg/mL and a greater dye removal has been observed as the olive pomace concentration increased. In the fixed bed adsorption experiment performed at bench scale, a significant increase in methylene blue uptake was observed using a mixture of olive pomace and charcoal in a multi-layer packed column. OMSW has also been used to remove heavy metals from industrial wastewater. Chouchene et al. (2014) reported a removal efficiency of Cu and Ni of 3.6 and 1.7 mg/g, respectively using OMSW, while Anastopoulos et al. (2015) showed that olive by-products could be effectively used to remove Pb and Cd at a pH range of 5–6, but less efficient in removing Cu, Cr, Zn, and particularly Ni.

The adsorption capacity of OMWs in removing some pollutants could be, however, not satisfactory in some cases due to their lower specific surface area and porosity. A modification of OMWs to improve their textural properties and adsorption capacities is then necessary. Thermochemical treatments, including pyrolysis, HTC, gasification, and torrefaction, are valid routes to convert biomass into renewable carbon materials with high adsorption capacities (Enaime et al. 2020b). Activated carbon (AC) derived from OMWs has been largely used as an adsorbent to remove organic and inorganic contaminants with a simple operating design (Hazzaa and Hussein 2015; Berrios et al. 2012). The preparation method and the experimental conditions affect significantly the final textural properties of the produced AC and hence its adsorption capacity. Enaime et al. (2017) explored the adsorption of indigo carmine dye from aqueous solution onto ACs prepared from OMSW impregnated with OMWW by chemical activation using potassium hydroxide and phosphoric acid as activation agents and physical activation using steam. The prepared ACs showed high removal efficiencies towards indigo carmine dye with that prepared by KOH chemical activation showing the highest adsorption capacity due to its higher micro- and mesoporous volume and its larger specific surface area. Other investigations also reported on the effectiveness of AC derived from exhausted olive-cakes (Baccar et al. 2009), olive peel and seed (Petrella et al. 2018), and raw olive stones (Hodaifa et al. 2013a) in removing heavy metals such as Pb^2+^, Cu^2+^, Fe^3+^, and Cr^6+^ from aqueous solutions.

Even if OMWs are an available biomass, the cost of their conversion into AC and the optimization of the preparation process to design an AC dedicated to the adsorption of a target molecule still limit its wide application on an industrial scale. Recently, several studies have highlighted the capacity of biochars and hydrochars as eco-friendly and low-cost alternative materials to successfully adsorb heavy metals, dyes and other organic pollutants from water and wastewater (Abdelhadi et al. 2017; Delgado-Moreno et al. 2021; Saleem et al. 2019). A concise review on different thermal conversion technologies and applications of char in wastewater treatment has been provided by Enaime et al. (2020b). The adsorption capacity of chars is strongly affected by the composition of the starting biomass, the production method, and the processing conditions, with temperature playing the key role. Abdelhadi et al. (2017) tested the adsorption efficiency of biochars produced from two- and three-phase OMSW from two different olive cultivars (Picual and Souri). Two-phase olive mill wastes derived from Picual cultivar and carbonized at 350 °C exhibited the best adsorption capacity. Authors suggested that even the biochar showed lower surface area (1.65–8.12 m^2^/g) as compared to commercial AC (1100 m^2^/g); its adsorption capacity towards Cu^+2^, Pb^+2^, Cd^+2^, Ni^+2^, and Zn^+2^ was more than 85% higher, suggesting that the surface area cannot be used as a sole predictor of heavy metal removal capacity and that other mechanisms related to the presence of functional groups on the surface of biochar could interfere. Izghri et al. (2020) proceeded to the preparation of hydrochars from TPOMW impregnated with FeCl_3_, which subsequently served as catalysts in advance oxidation process for the removal of methylene blue from aqueous solutions. According to Izghri et al. (2020), HTC of TPOMW at 250 °C during 4 h using a FeCl_3_ to TPOMW ratio of 1.5 allowed producing hydrochar with high mass yield (66%) and promising performances as a catalyst in heterogeneous Fenton-Like oxidation achieving 91% of methylene blue removal. The produced catalyst can be reused for successive cycles as it showed high stability and very less iron leaching properties. Delgado-Moreno et al. (2021) used pyrolysis and HTC to convert OMWs (OS, olive tree pruning, and pitted and reprocessed wet OMSW) into chars, which have been then used together with commercial biochars and a commercial AC as adsorbents to remove triclosan, ibuprofen and diclofenac from water. Despite of its low surface area (7.5 m^2^/g), hydrochar produced at 240 °C exhibited higher adsorption capacities (64% for diclofenac, 43% for ibuprofen and 98% for triclosan) due to its acidic pH and its surface rich in oxygenated functional groups.

Within the context of the circular economy, the use of OMSW in integration systems has also been proposed for the removal of polyphenols from OMWW. Allaoui et al. (2021) reported an adsorption capacity of about 381 mg/g of polyphenols from crude OMWW using crude OS as an adsorbent. Similarly, Stasinakis et al. (2008) tested the adsorption capacity of three different types of olive pomace, namely dried olive pomace, dried and solvent extracted olive pomace, and dried, solvent extracted, and incompletely combusted olive pomace. According to the results, dried, solvent extracted, and incompletely combusted olive pomace at a concentration of 10 g/L was able to remove more than 40% of polyphenols having an initial concentration of 50 mg/L, while showing a better stability as compared to the two other samples tending to release polyphenols. Authors also reported that fixed bed sorption experiments with lower flow rates and smaller particle size of sorbent resulted in longer column exhaustion time and higher initial removal efficiency. In another study, Enaime et al. (2019) successfully used OS as a filter media for the pretreatment of OMWW, achieving COD and polyphenols removal efficiencies of about 23.2% and 11.3%, respectively. Esteves et al. (2022) proceeded to the activation of olive stones and wood from olive tree pruning by physical (CO_2_) and chemical (KOH) methods for their subsequent use to remove OMWW phenolic compounds. Results showed that chemically activated olive stones present better performance (200 mg/g) than the physically activated sample (189 mg/g), due to its improved surface area and microporosity. Nevertheless, while the S_BET_ increases from 792 to 1013 m^2^/g (i.e., ca. 28%), phenolic compound removal only improved by 5.8%, which is also an indicative that additional parameters should be considered. Authors also reported the possible thermal regeneration of the saturated adsorbents, while maintaining their performances. In another study, Abid et al. (2022) investigated the conversion of OMSW into a biochar that was used as an adsorbent for the removal and the recovery of polyphenols from OMWW. A maximum polyphenol adsorption of 140.5 mg/g was achieved with a high affinity of hydroxytyrosol to be recovered due to the polar nature of biochar surface. The adsorption capacity of biochar towards phenolic compounds is significantly influenced by the biochar surface charge, its surface area and pH and the abundance of carboxylic and lactonic functional groups. Hanandeh et al. (2021), while testing the efficiency of biochars derived from OMSW previously treated by FeCl_3_ and pyrolyzed at temperature of 550 °C for the removal of phenolic compounds from OMWW, achieved an adsorption capacity of about 103.9 mg/g at a pH of 2. Authors suggested that chemisorption is the dominating mechanism in the adsorption of phenolic compounds on biochar and that even the adsorption on the pre-treated biochar was 33% higher than the post-treated biochar; it is more economical to use post-treatment of the produced biochar with FeCl_3_ than the pretreatment of the starting biomass, as it reduces the use of FeCl_3_ by 75%.

### OMWs as a source of bioactive components

The widely known benefits of olive oil is mainly due to their high content of bioactive molecules, which make it a product widely demanded by the consumer. These bioactive components are detected not only in olive oil but also at significant levels in its processing by-products, including OMWW and OMSW (Parkinson and Cicerale 2016). From these by-products, different bioactive molecules such as fatty acids, phenolic compounds, phytosterols, triterpenoids, tocopherols, and coloring pigments (chlorophylls and carotenoids) could be recovered. Some other bioactive compounds, like carotenoids or chlorophylls, have also been detected in olive oil extraction by-products but in low quantities (Hannachi et al. 2020; Otero et al. 2021). The concentration of bioactive molecules and their distribution in olive by-products varies depending on many factors including growth and climatic conditions, variety and maturity of olives, olive oil extraction method, and the by-product considered and its freshness (Romero et al. 2018). The storage of OMWs is a parameter that significantly affect their composition. For instance, an accumulation of hydroxytyrosol and a reduction of other monomeric and oligomeric phenolic components were reported as a result of the prolonged storage of OMWW (Feki et al. 2006).

Recently, more attention has been attributed to the reintegration of bioactive compounds recovered from OMWs in food and pharmaceutical industries and extensive studies have been performed to elucidate their chemical and biological properties and their biological properties. The different biological activities of OMWs-derived bioactive compounds, including antioxidant, anti-inflammatory, anticancer, and other activities, and the associated mechanisms were deeply reviewed by Otero et al. (2021). Biophenols constitute one of the major groups of bioactive compounds present in OMWs that are reported responsible for many biological activities. It is assumed that about 98% of the phenolic compounds present in olive fruit find their way to olive wastes, either in OMWW (approx. 53%) or in OMSW (approx. 45%) (Rodis et al. 2002). These hydrophilic components are mostly represented by phenyl alcohols, phenolic acids, secoiridoids, and flavonoid groups. Hydroxytyrosol, which is the main phenolic compound detected in OMWW, together with other simple phenols and flavonoids are reported for their high antioxidant activity, in addition to other activities such as cardioprotective and cancer preventing activities (Obied et al. 2005). Other bioactive compounds highly abundant in OMWs are oleosidic compounds resulting from the hydrolysis of oleuropein and its derivatives; it is assumed that about 80% of oleuropein contained in olive fruit is degraded during the olive oil extraction following the operations of crushing and malaxation (He et al. 2012). Oleosidic compounds and its derivatives are showing an important antibacterial activity, even higher than the well-studied oleuropein and many other simple phenols (Medina et al. 2007).

The diverse biological activities of OMWs bioactive compounds and their useful properties have created an interest in their extraction. The feasibility and efficiency of the recovery method are, however, the key factors for using these compounds as alternative natural antioxidants and antimicrobials. Several methods have been reported for the recovery of bioactive compounds from OMWs (Table 3). Liquid–liquid solvent extraction using organic solvents is typically the widely used technique for the extraction of bioactive compounds from different matrices. The organic solvents used provide a physical carrier for target compounds to be transported between different phases and then be recovered (Galanakis 2012). The nature of the solvent is an important factor affecting the extraction efficiency. Methanol–water mixtures have been effectively used for the extraction of phenols with high yields and widest arrays, while flavonoid aglycons have been effectively extracted using ethyl acetate (De Leonardis et al. 2007; Sannino et al. 2013). Other polar protic mediums like hydroalcoholic mixtures have been also used at different concentrations for the extraction of phenolic acids. The industrial application of bioactive compounds recovered by liquid–liquid extraction method could be however limited due to the toxicity and the inedibility of some solvents, raising then environmental, health, and safety concerns (Galanakis and Kotsiou 2017). Alternatively, bioactive compounds could be recovered by membrane filtration systems including micro-filtration, ultrafiltration, nanofiltration, and reverse osmosis. Servili et al. (2011a) proceeded to the recovery of hydrophilic phenols from fresh olive vegetable water in an industrial plant using a three-phase membrane system (microfiltration, ultrafiltration, and reverse osmosis) prior enzymatic treatment. This approach yielded a phenolic compounds-enriched concentrate that was effectively used for enriching the antioxidant content of virgin olive oil. Natural-based filters such as starch filters extracted from fruits, cereals, and tubers have also been used by Fernandez-Gutierrez et al. (2013) in a patented process for the extraction of bioactive compounds from olive oil by-products. Phenolic compounds were also separated from OMWs using different adsorbents such as AC and resins. For instance, Yangui and Abderrabba (2018) extracted total phenols (75.4%) and hydroxytyrosol (90.6%) from OMWW using AC coated with milk proteins. A high extraction yield (90%) of hydroxytyrosol from olive leaves has also been conducted using modified AC in batch and column systems through adsorption and desorption processes (Hadrich et al. 2022). Rubio-Senent et al. (2012) proceeded to the extraction of phenolic compounds by hydrothermal treatment of OMWs. This patented process promoted the breakdown of oleuropein, dimethyloleuropein, and verbascoside, which resulted in higher concentrations of hydroxytyrosol. Similarly, Lama-Muñoz et al. (2014) proceed to the recovery of antioxidant compounds from liquid fraction (prehydrolyzates) issued from autoclave treatment of olive stones at 130 ºC for 90 min followed by a dilute acid extraction (2% w/v sulfuric acid) to recover pentose sugars.

**Table 3:** Bioactive compounds recovered from the different by-products of olive oil industry, their relative percentage, and their extraction methods

Compounds	Originated by-product	Relative concentration	Extraction method	Reference
Phenolic compounds	OMWW	432.59 ± 44.63 mg gallic acid/L	Liquid–liquid extraction and Fenton’s process integration	Nunzioa et al. (2018)
	Olive vegetation water	588.8 ± 2.3 mg/kg	Membrane filtration liquid/liquid extraction	Martins et al. (2021)
	Olive pomace	0.69 ± 0.06 mg gallic acid equivalent/mL	Water extraction	Hannachi et al. (2020)
	Olive stones	761.83 ± 1.97 mg gallic acid equivalent/100 g DW	Maceration process using methanol as extraction solvent	Servili et al. (2011a)
	Olive leaves	211.385 mg tannic acid equivalent/g	Methanol extraction	Rahmanian et al. (2015)
Hydroxytyrosol	Olive stones	24.29 ± 0.48 mg/kg	Solid–liquid extraction using (methanol, ethanol, and acetone) as solvent	Nakilcioğlu-Taş and Ötleş (2019)
	Olive leaves	0.53–1.12% of dry olive leaf	Ethanol extraction	Guinda et al. (2015)
	OMWW	31 ± 0.2 mg/kg	Pectinases treatment and membrane filtration	Troise et al. (2014)
	OMWW	7.1 ± 0.1 mg/kg	8/2 MeOH–water (v/v) solution	Benincasa et al. (2019)
	Olive tree biomass from pruning	0.18 ± 0.01 mg/g	Supercritical fluid extraction	Benincasa et al. (2019)
	Olive pomace	16.0 ± 0.2 mg/kg	8/2 MeOH–water (v/v) solution	Caballero et al. (2020)
	Alperujo	0.111 ± 0.02 mg/g	Ultrasound-assisted extraction and natural deep eutectic solvents	Plaza et al. (2020)
Tyrosol	Olive pomace	1.9 ± 0.1 mg/g	Pectinases treatment and membrane filtration	Troise et al. (2014)
	OMWW	13.98 ± 0.04 μg/mg	Liquid–liquid extraction and Fenton’s process integration	Martins et al. (2021)
	OMWW	29.94 mg/L	Methanol extraction	Bruno et al. (2021)
	Olive stones	1–8 mg/g dry tissue weight as tyrosol	Methanol:water extraction	Ryan et al. (2003)
	Olive cake from two phases process (Argentina)	1.35 × 10^−3^ ± 4.8 × 10^−5^ mg/g	Ultrasound-assisted extraction and natural deep eutectic solvents	Plaza et al. (2020)
Oleuropein	OMWW	9.67 mg/L	Methanol extraction	Bruno et al. (2021)
	Pomace	22.47 mg/g	Methanol extraction	Bruno et al. (2021)
	Olive leaves	218 ± 11 mg/g / 37.8 ± 2.0 mg/g	Supercritical fluid extraction/Soxhlet extraction	Plaza et al. (2020)
	Olive pomace	7.16 ± 0.19 mg/g	Supercritical fluid extraction/Soxhlet extraction	Plaza et al. (2020)
	Olive stones	33.22 ± 0.38 mg/kg	Solid–liquid extraction using methanol, ethanol, and acetone as solvent	Nakilcioğlu-Taş and Ötleş (2019)
Syringic acid	Olive stones	0.68 ± 0.01 mg/kg	Solid–liquid extraction using methanol, ethanol, and acetone as solvent	Nakilcioğlu-Taş and Ötleş (2019)
p-coumaric acid	OMWW	9.00 ± 1.40 μg/mg	Liquid–liquid extraction and Fenton’s process integration	Martins et al. (2021)
	OMSW	32 ± 2 mg/L	High-temperature thermal pre-treatment and phenol recovery using an industrial chromatographic system	Serrano et al. (2017)
Syringic acid	OMWW	14.00 ± 1.41 μg/mg	Liquid–liquid extraction and Fenton’s process integration	Martins et al. (2021)
Vanillic acid	Pomace	5.83 mg/L	Methanol extraction	Bruno et al. (2021)
	Olive tree biomass from pruning	2.10 mg kg	Methanol extraction	Bruno et al. (2021)
	Olive leaves	0.01 ± 0.002 mg/g	Supercritical fluid extraction	Caballero et al. (2020)
Vanillin	OMWW	4.14 mg/L	Methanol extraction	Bruno et al. (2021)
	Pomace	3.65 mg/kg	Methanol extraction	Bruno et al. (2021)
	Olive tree biomass from pruning	0.60 ± 0.07 mg/g	Supercritical fluid extraction	Caballero et al. (2020)
Luteoin-7-O-glucoside	OMWW	11.89 mg/L	Methanol extraction	Bruno et al. (2021)
	Pomace	7.95 mg/kg	Methanol extraction	Bruno et al. (2021)

The emergent interest in the recovery of bioactive compounds increased the need to develop more efficient extraction technologies in order to reduce solvent consumption, shorten processing time, increase recovery yield, improve product quality, and enhance functionality of extracts, while lowering energy consumption as compared to conventional extraction methodologies (Rosello-Soto et al. 2015). New extraction technologies such as microwave assisted extraction, ultrasonic-assisted extraction, pressurized liquid extraction as well as electrotechnologies including high-voltage electrical discharges, ultrasound-assisted extraction, and others have also been introduced (Barba et al. 2015; Rosello-Soto et al. 2015; Galanakis 2021; Otero et al. 2021). In addition to being highly efficient, requiring low-energy consumption and short extraction time, these emerging technologies have the advantage of not involving high temperatures, which avoids damaging the structure of the extracted compound and helps preserving their bioactive content (da Rosa et al. 2021). Gómez-Cruz et al. (2021) proceeded to the extraction of phenolic compounds from exhausted olive pomace by microwave-assisted method using water as extraction solvent under different temperatures, extraction times, and solid loading conditions. The microwave-assisted extraction method at 99.7 °C, 3.9% (w/v) solids, and 34.3 min allowed for a maximum content of hydroxytyrosol (6 mg/g of exhausted olive pomace), which makes of the extract a potential alternative to be used as a functional and antioxidant additive. In another study, Niknam et al. (2021) used ultrasound-assisted extraction to extract phenolic compounds from dried and defatted olive pomace using 50% v/v ethanol–water as solvent. The extraction process yielded 14.70 mg/g total phenols, 2.48 mg/g total flavonoids, and 0.924 mmol Trolox/g antioxidant activity. A further treatment step using polymeric resins and activated charcoals followed by a desorption step using acidified ethanol–water has been used for the purification of the extracted biophenols. The overall process allowed a recovery of total phenols, hydroxytyrosol, and tyrosol of about 57.65%, 19.27%, and 45.73%, respectively. Other sustainable alternatives have also been studied. Sklavos et al. (2015) used solar distillation to simultaneously dry and recover antioxidant compounds from OMWW distillate. This system allowed, however, the recovery of only 4% of the initial phenols, with tyrosol present in all samples and hydroxytyrosol only in freshly collected samples. Further studies are then required to improve these findings.

Due to their antioxidant and antimicrobial properties, various applications of bioactive compounds derived from OMWs have been suggested (Table 3). The application of phenols in food industry is a promising alternative to mitigate the challenges associated to health problems related to the excessive use of synthetic antioxidants. By introducing olive leaves extracted phenols, mainly hydroxytyrosol and oleuropein aglycone, in refined olive oil and olive pomace oil, Bouaziz et al. (2010) reported a significant increase in the resistance of oils to oxidation. In the study of Lafka et al. (2011), OMWs-derived extracts were introduced in virgin olive oil and sunflower oil. Although its phenolic yield was not quite high, the tested extracts showed stronger antioxidant capacity than that observed by using BHT, ascorbyl palmitate, and vitamin E. In another study, Troise et al. (2014) reported that the addition of OMWW phenolic powder to ultra-pasteurized milk was able to trap the reactive carbonyl species such as hydroxycarbonyls and dicarbonyls and inhibit the formation of Maillard-derived off-flavor compounds during UHT treatment, which improved the nutritional and sensorial qualities of the milk. An extract from olive pomace was also used to substitute sulfur dioxide usually added to wine as a preservative and to prevent microorganisms’ proliferation (Ruiz et al. 2017). Exhibiting similar structures and mechanisms of action as synthetic compounds, OMWs-derived bioactive compounds, such as monounsaturated fatty acids, minerals and phenolic compounds could be applied in several pharmaceutical and cosmetic applications (Rodrigues et al. 2017). Their integration in creams, balms, shampoos, or hair conditioners was frequently used. Antioxidants such as hydroxytyrosol, oleuropein, caffeic acid, and flavonoids, are widely reported for their ability to mitigate the effects of skin ageing process, to scavenge oxidative substances and to act as UV blockers. The efficient application of phenolic compounds in cosmetic applications could be, however, limited due to their character of being very soluble in water, which promote their removal from the skin during seawater immersion.

Despite the high number of studies carried out for the recovery of bioactive compounds from OMWs and their reuse in different applications and the promising results obtained, most of them have been focused on the recovery of phenolic compounds. More research studies investigating the recovery and application of other bioactive compounds, such as monounsaturated fatty acids, are still needed.

### Other applications of OMWs

OMWs have also attracted the building materials industry as low cost and available raw materials that can be used to replace or to be mixed with commercial products. OMWW issued from three-phase and two-phase olive oil extraction were used to replace freshwater in the manufacture of fired clay bricks (Mekki et al. 2006b). De la Casa et al. (2009) reported that the use of two-phase OMWW instead of freshwater in clay brick manufacture allowed the production of bricks with similar technological properties and extrusion performance as freshwater-based ones, with a simultaneous decrease in the heating requirement in the range of 2.4–7.3%. Incorporating OMWs into brick manufacturing is not only a promising disposal route for olive mills but can also bring economic benefits to the building materials industry by reducing the heat required during the brick production process. The water contained in OMWs is returned to the atmosphere during the drying of brick before their firing, while the solid matter remains locked in the bricks, which provides supplementary heat to the kiln during the firing stage (Mekki et al. 2006b). Silvestri et al. (2021) evaluated the technical feasibility and the environmental and economic sustainability of integrating OMWW in the fired clay brick production. Authors reported a decrease in global warming potential and in fossil fuels depletion of up to 3.1% and 4.3%, respectively, while no significant variations for the toxicity impact category were observed. Authors also reported that a beneficial use of OMWW in fired clay brick process should be associated to the installation of olive oil mills in a distance of less than 150 km from the brick factory (Silvestri et al. 2021). Several advantages have also been reported following the incorporation of olive pomace as an ingredient in ceramic manufacturing as compared to conventional ceramic products, including a 10% lower density and 18% lower thermal conductivity, in addition to an energy savings due to their organic matter content (Ruiz et al. 2017). Other application of OMWs have been also studied. The high concentration of OMWs in minerals, fatty acids and bioactive compounds, characterized by their moisturizer and antiaging properties, promotes their application in spa treatment (Rodrigues et al. 2017). OMWs could also be used as liquid growth medium for lipolytic microorganisms due to their content in residual oil that vary depending on the olive oil extraction process (Asses et al. 2009). Dye industry is another field of application of OMWs. OMWs characterized by their typical color could be a valuable source of natural dyes, which can serve as alternative synthetic dyes. Some research studies have been done to define the optimum conditions for the extraction of dyes from olive pomace with promissory results, while others studied the applicability of dyes derived from OMWs in textile industry (Meksi et al. 2012).

## Issues related to OMWs management and barriers of the possible implantation of the proposed solution

Previously, OMWs treatment was not compulsory; thus, the most common and cheapest method of dealing with the large quantities of annually produced OMWs has been its disposal in open evaporation ponds or its draining in the environment at no cost for OMWW and its use as biofuel in boilers for OMSW, even the obtained energy yields are not satisfying. The absence of any obligation for olive mills to bear the disposal costs associated to the treatment of OMWs makes management methods based on resource recovery not attractive. However, the growing social awareness of environmental issues exerted pressure on governments to impose stricter laws and regulations in order to make OMWs treatments compulsory and preserve ecosystems. Besides the negative impact of the large produced amounts of hazardous by-products, olive oil production process is also criticized for its high energy consumption. The consideration of these two aspects makes the application of waste-to-resource concept in olive sector a promising solution. Olive mill solid and liquid wastes contain a significant energy content that can be recovered in different forms, 1 cm^3^ of OMWW could produce 60–80 kWh of energy in the form of biogas, while 1 kg of OMSW could be converted into 18,000 kJ of heat (Schmidt and Knobloch 2000). OMWs treatment aiming at energy recovery can therefore represent an interesting and sustainable alternative to partially meet the energy needs of oil mills. Moreover, OMWs could be used as additive in agricultural sector, as an adsorbent material for wastewater treatment, or as an attractive source of bioactive compounds.

Despite the large number of investigations having dealt with this topic, which in some points reported different findings, the opinion of the scientific community concerning the potential of OMWs as promising resources and the advantage of their optimal valorization is common. Though, several restrictions are still limiting the competitivity of the OMWs-derived resources and the scalability of the so far suggested solutions. The capability of OMWs-derived resources to create a positive economic balance is conditioned by the availability of the waste, the in-depth knowledge of its variable composition, and the simplicity and efficiency of the recovery technology. Moreover, the small dimensions of most olive mills and their spread-out distribution in the territory, in addition to their seasonal operation (limited to the period from November to February), make the calculation of the average annual waste production very uncertain and hamper the feasibility of larger centralized treatment plants and their continuous operation. Moreover, depending on the adopted trituration process, the olive variety, and the duration of storage, a high variety in OMWs properties could be expected, which make it impossible to apply a unique solution for their management. Taking all these variables into account makes it complicated to decide for a valuation method. The decision becomes more difficult considering that each method has its own advantages and disadvantages, and depending on the needs of the operator, the appropriate method may be different. For instance, although biological processes aimed at producing bioenergy from OMWs are mentioned as less expensive than other processes requiring complicated technologies, a great mastery of the treatment process is necessary, as it brings into contact microorganisms with phenolic compounds and lignin (in OMSW), known for their recalcitrant and antimicrobial properties. This makes the process start-up a critical phase that requires special attention. The complication of OMWs matrix, the presence of toxic compounds, and the potential formation of inhibiting intermediates could also reduce the production of biogas (Neshat et al. 2017). Concerning the safety of biogas production units, it is reported that biogas becomes flammable and can cause explosions when it comes into contact with O_2_. All these aspects require continuous control and monitoring of the process to avoid any risk of instability or unsafe situations, thus resulting in additional operating costs. Moreover, long retention times are generally required during anaerobic processes, which imply the need for large reactor volumes. This could be economically not attractive for olive mill operators. Hence, despite the technological progress achieved in using biochemical processes for energy production, the technology itself, when used for OMWs treatment, is still considered not mature yet to attract investors and be scaled up (Nunes et al. 2020). This makes the thermal processes more attractive. While adding OMWW to OMSW is counterproductive from the energy conversion point of view for dry thermal processes, it is advantageous for HTC processes. When comparing the energy saving of HTC and pyrolysis during the thermal treatment of OMWs with higher moisture content, it seems that HTC could be a promising method to convert OMWs into biofuel, with an energy saving of about 49% (Enaime et al. 2022). This is also beneficial for organizational and economic considerations, as no separate wastewater disposal will be required. Nevertheless, HTC process also requires optimization especially that HTC reactions are occurring at relatively high pressure. In addition, the process generates a liquid phase that could be considered as a concentrate of acids and different compounds, requiring additional treatments.

There is definitely a big need to explore the versatile bioactive compounds derived from OMWs and their treatment by-products and to introduce them in pharmaceutical, cosmetics, or even food industries, especially with the increasing interest of consumers for natural products. Even though the versatile composition of bioactive compounds derived from OMWs and their biological activities are very well known and their safety is also documented, the awareness of olive farmers on the importance and the potentialities of these compounds remains, however, very limited (Rodrigues et al. 2015). Olive farmers are the suppliers, and their involvement will guarantee the regular supply of interested industries. Moreover, the implementation of the policies is mainly focused on applications that mainly support bioenergy and biofuel production. One important reason behind this is the challenges related to economic aspects, as the recovery of bioactive compounds and their industrial application are mainly associated with high capital costs and complicated technologies that small olive oil industries could not support. In addition, considering environmental and health aspects, more sustainable extraction methods with reasonable investment should be adopted to reduce excessive use of potentially toxic solvents.

As summarized in Table 4, each OMWs valorization method has its specific limitations, but the most important limiting factor is the lack of government and community involvement and the absence of subsidies for investments to support the implementation of the proposed strategy. Although the various economic, social, and environmental benefits of waste recovery are recognized, there is still no practical support for this concept.

**Table 4:** Main advantages and limitations related to the valorization of OMWs and their derivative by-products

Valorization method	Advantages	Limitations
Energy generation	-Replaces fossil fuel as an energy source -Contributes to the optimization of natural resources’ use -Contributes to minimize GHG emission -Provides an extra income to farmers and olive-mills` operators -Creates new source/ opportunities of employment and enhances the social fabric of rural and agricultural areas -Contributes to the national GDP (gross domestic product)	-Cost requirement to collect and transport OMWs from mills or farms to the utilization facilities -High operation and overhead cost that could impact the economically benefits -Toxicity of OMWs causing process stability problems during biological conversion methods: Required pre-treatment is not cost-effective and could affect negatively on the efficiency of energy recovery process -Lack of resources (technological, financial, and innovation challenges) -Environmental impacts of energy recovery process (digestate, HTC liquid effluent) in case of unrestricted long-term management plan
Agriculture applications	-Great potential of OMWs to improve soil fertility and soil organic matter: high content in valuable plant nutrients (nitrogen, phosphate, potassium, iron, and magnesium) -Slowing down soil-erosion processes specially in hilly areas -Improve the structure of soil aggregates and consequently increase soil porosity and water retention capacity -Supports the sustainability of the agricultural sector -Increase land yield -Strengthen and extend sorption of insecticides and herbicides, thus reducing their biodegradation, slow down their leaching, which reduces groundwater pollution risk	-Some chemical components in OMWs might result in soil and water pollution, in addition to the risk of phytotoxicity -Raw OMWs application to soil could deteriorate the oxygen uptake efficiency -Constraints related to the availability of OMWs, investment costs and the industrial or agronomic environment -Local regulations and laws could restrict the optimum benefits of OMWs applications -Lack of long-term sustainability strategies
Extraction of bioactive compounds	-Support green chemistry principles -Minimize environmental source depletion -Contributes to reducing chemicals demand and minimize environmental impacts related to chemicals ingredients production cycle	-Generates solvent waste -The application of bioactive compounds extracted from OMWs are subjected to restricted regulations and requirements, which could be considered as a challenge -Limited market demand -Technical limitations that include but not limited to substrate seasonal storage at ideal conditions and bioactive compounds extraction -Stability of extracted bioactive compounds during the utilization cycle
Construction applications	-Minimizes the pressure on freshwater sources -Reduces the overall process costs by replacing conventional water with unconventional sources with a lower cost per litter -Minimizes carbon footprint associated to construction industry -Lightweights’ construction material that leads to minimizing economic and environmental impacts -Good insulating characteristics that reflect on the energy consumption at purpose of heating/cooling -Reduces the heat required during the ceramic production process, resulting in lower greenhouse gas emissions from production line	-Cost requirement to collect and transport OMWs from mills or farms toward the utilization facilities

Based on the insights discussed above, the following policy recommendations, which involve all stakeholders, can be proposed:

- Classification of olive oil extraction industries according to their size and waste production capacity in order to implement specific measures for the management of OMWs; separate facilities within the industry itself or creation of cooperatives at regional level for small olive mills.
- Carrying out interviews with various stakeholders and field visits in order to analyze all political, legal, economic, social, geographical, and technical factors and create a database describing the specific need of each region.
- Support scientific research and development, which can provide the fundamental bases of technological innovation in the field of OMWs valorization allowing to achieve satisfactory results.
- Development of a network between scientists to facilitate communication and strengthen cooperation between them, which could avoid the inutile waste of time, resources, and expertise.
- Establishment of specific policies to help olive oil producers and cooperatives adopt sustainable waste management practices; for instance, promoting dialogue with scientists, this will allow multiple stakeholders to be connected to research and entrepreneurial projects dedicated to OMWs valorization.
- Governmental support of the waste-to-resources strategy in the olive sector through investment incentives and sustainable partnerships between public and private sectors.

## Conclusion and prospects

Olive sector is committed to environmental obligations because of their unused high amounts and environmental harmful wastes. The growing awareness of the social community coupled with the legislative obligations has put increasing pressure on the olive industry to adopt new alternatives for the sustainable management of their wastes and byproducts. The implementation of the waste-to-resource concept will avoid the negative impact of OMWs, while producing new alternative resources, allowing the producers to diversify their activities and increase their capital. The data collected in this review shows that OMWs exhibit promising valorization potential from which different resources (biogas, biochar, activated carbon, bioactive compounds, soil conditioner, fertilizer, additive in construction materials…) could be recovered and reused in several sectors such as energy sector, agriculture, wastewater treatment, food and pharmaceutical industries, and construction industry. Although, this scientific evidence, a rational approach to OMWs valorization, has not been fully implemented. Several technological, organizational, and social factors are still required to allow converting OMWs into competitive resources able to create a positive economic balance. These include encouraging the continuous exchange and collaboration between industrial sector, including small olive oil extraction units, policy makers, and scientific research unities, increasing consumers’ awareness of the waste-to-resource concept and their environmental and socio-economic benefits, and strengthening the support of state through investment incentives and sustainable partnerships between public and private sectors.

This review contributes to an overall understanding of the different technologies that can be used for OMWs valorization within the waste-to-resources concept. However, most of the reported research studies are conducted in laboratory or in pilot scale; the results of these tests are not easily correlated to large-scale applications. More studies focused on large-scale plants in long-term operations are needed for the safe transfer of the research and development efforts into the industrial avenue. It is also crucial to perform techno-economic assessment of the various valorization choices at industrial scale to give an overall projection of the available potency and understand various scale-up issues. As some of the technologies suggested for OMWs valorization, such as biological processes, require a previous pretreatment, the focus of future research should be on designing pre-treatment strategies that will be able to withstand the inherent heterogeneity of OMWs composition. Preventive mechanisms should also be developed to avoid the long storage of OMWs resulting in possible oxidative loss of some of their bioactive compounds. It is also important to direct scientific efforts toward establishing combined processes rather than individual treatments for the valorization of OMWs to allow the management of all waste streams, even those produced during the valorization process itself. There is also a big gap in the integration of kinetic and statistical models for the optimization of these combined processes. These models could be useful for describing experimental systems and scaling them up.

## Data Availability

Data will be made available on request.
